# Combined meta-analysis of preclinical cell therapy studies shows overlapping effect modifiers for multiple diseases

**DOI:** 10.1136/bmjos-2020-100061

**Published:** 2021-04-19

**Authors:** Peter-Paul Zwetsloot, Ana Antonic-Baker, Hendrik Gremmels, Kimberley Wever, Chris Sena, Sanne Jansen of Lorkeers, Steven Chamuleau, Joost Sluijter, David W Howells

**Affiliations:** 1 Experimental Cardiology, UMC Utrecht, Utrecht, The Netherlands; 2 Neuroscience, Monash University, Melbourne, Victoria, Australia; 3 Department of Neuroscience, The Alfred Central Clinical School Monash University, Melbourne, Victoria, Australia; 4 Medical Microbiology, UMC Utrecht, Utrecht, The Netherlands; 5 Systematic Review Centre for Laboratory Animal Experimentation, Radboud Universiteit, Nijmegen, Gelderland, The Netherlands; 6 Department of Clinical Neurosciences, Edinburgh Royal Infirmary, Edinburgh, UK; 7 Cardiology, UMC Utrecht, Utrecht, The Netherlands; 8 Cardiology, Amsterdam UMC, Amsterdam, Noord-Holland, The Netherlands; 9 School of Medicine, University of Tasmania, Hobart, Tasmania, Australia

**Keywords:** preclinical meta-analysis, ratio of means, cell therapy

## Abstract

**Introduction:**

Cell therapy has been studied in many different research domains. Cellular replacement of damaged solid tissues is at an early stage of development, with much still to be understood. Systematic reviews and meta-analyses are widely used to aggregate data and find important patterns of results within research domains.

We set out to find common biological denominators affecting efficacy in preclinical cell therapy studies for renal, neurological and cardiac disease.

**Methods:**

We used datasets of five previously published meta-analyses investigating cell therapy in preclinical models of chronic kidney disease, spinal cord injury, stroke and ischaemic heart disease. We transformed primary outcomes to ratios of means to permit direct comparison across disease areas. Prespecified variables of interest were species, immunosuppression, cell type, cell origin, dose, delivery and timing of the cell therapy.

**Results:**

The five datasets from 506 publications yielded data from 13 638 animals. Animal size affects therapeutic efficacy in an inverse manner. Cell type influenced efficacy in multiple datasets differently, with no clear trend for specific cell types being superior. Immunosuppression showed a negative effect in spinal cord injury and a positive effect in cardiac ischaemic models. There was a dose–dependent relationship across the different models. Pretreatment seems to be superior compared with administration after the onset of disease.

**Conclusions:**

Preclinical cell therapy studies are affected by multiple variables, including species, immunosuppression, dose and treatment timing. These data are important when designing preclinical studies before commencing clinical trials.

**Strengths and limitations of this study**

- Direct comparison of different research fields through meta-analytical use ratio of means.
- Outcomes of preclinical cell therapy studies in multiple fields are influenced by experimental choices like animal size, dose and timing of the intervention.
- Since this is a new analysis on existing datasets, a systematic new search was not performed.
- There is no prepublished analysis protocol.

## Introduction

Stem and progenitor cells have emerged as potential therapies in many different areas of medicine. They have the capacity to replace or stimulate repair of damaged tissue, can be used to study human development and disease, and serve as a test bed for discovering new drugs and gene therapies. Cellular products as a therapeutic have provided new paradigms of regeneration for many organs, especially organs that do not heal easily, such as the brain, like the brain, heart, kidney, cartilage and eye.^1^ In light of the overwhelmingly positive results seen in preclinical studies, cell therapy is being translated into the clinic in multiple research fields. The rationale for transplanting these stem and progenitor cells is bifold: potentially replacing lost tissue and predominantly supporting the surviving cells through paracrine mechanisms or modulation of the immune response.^2 3^ Study of these mechanisms points towards soluble growth factors, cytokines and extracellular vesicles as major mediators in these processes.

Preclinical studies are often the starting point for such promising new therapies. Animal experiments allow exact control of experimental conditions and access to postmortem material with fewer restrictions than human trials, while maintaining the complexity of a whole organism. Preclinical models of disease have been standardised to a large extent and are ideally comparable across research centres. Rodent models are most frequently used, as rodents are easy to handle, are cost-effective to maintain, have a short generation span and the availability of inbred strains theoretically allows for great experimental reproducibility and stable breeding of genetically modified animals. On the other hand, it is argued that large animal models show greater similarity to human physiology. Studies in, for example, pigs are therefore often used as an intermediate essential ‘confirmatory step’ in the translational axis towards human application.^4^

In the preclinical application of cell therapies, multiple meta-analyses have been performed on studies using models of renal,^5^ neurological^6 7^ and cardiac disease models.^8 9^ Since the paracrine mode of action is hypothetically similar for these stem cell and progenitor cell injections, common effects and effect modifiers for these and future cell therapy research fields could be present in these datasets. In this paper, we focus on potential common denominators in these studies (eg, animal size, cell origin, cell type and immunosuppression) to look for certain overlapping cell therapy characteristics across disease entities. Subsequently, we searched for all phase III trials in these diseases, to see which research fields are getting close to clinical application.

## Methods

We used the original data of five previously published preclinical systematic reviews containing meta-analyses on the effect of stem and progenitor cell therapy in chronic kidney disease (CKD),^5^ stroke,^6^ spinal cord injury (SCI)^7^ and myocardial infarction (MI).^8 9^ The CKD dataset comprises all controlled studies (identified by searching PubMed and EMBASE in January 2014) reporting the efficacy of cells or cellular products in animal models of CKD. Primary outcomes were blood pressure, urinary protein, plasma creatinine, plasma urea and glomerular filtration rate. For the present analysis, we prespecified blood pressure and urinary protein as our outcomes. The stroke dataset comprises all controlled studies (identified by searching PubMed, EMBASE, BioSIS and ISI Web of Science in October 2009) reporting the efficacy of allogenic or autologous stem cells in animal models of focal cerebral ischaemia expressed as a change in structural (infarct size) or neurobehavioural outcome. Studies using interventions to mobilise endogenous stem cells were excluded. The SCI dataset comprises all controlled studies (identified by searching PubMed, EMBASE and ISI Web of Science in December 2011) reporting the efficacy of 45 different stem cell types in animal models of SCI induced by transection, hemisection, compression or contusion with outcome assessed by change in motor or sensory behaviour. This was the only dataset to rely solely on behavioural (motor and sensory) outcomes reflecting normal reporting standards for this field where lesion volume is difficult to delineate. The MI datasets included both acute and chronic models of ischaemic cardiomyopathy. One dataset^8^ included all animal studies in large animals (identified by searching PubMed and Embase in January 2013) that used cell therapy (myoblasts, bone marrow cells, mesenchymal stem cells and cardiac stem cells (CSCs)) in comparison to a control group. Primary outcome studied was ejection fraction (EF). The other MI dataset^10^ studied all animal models (identified by searching PubMed and Embase in October 2015) using CSCs in comparison to a placebo-controlled group (PBS/vehicle/other cells). The primary outcome studied was EF.

For the CKD, stroke and MI datasets, structural outcomes were reported as continuous variables. Behavioural outcomes were described by a number of ordinal scoring systems specific for the field as described in detail in each paper. We converted all outcome measures to ratios of means (ROMs) and its SE, based on the following formula’s (see Friedrich *et al* for detailed rationale and calculations).^11^

$$ROM=\frac{Mean_{control}}{Mean_{exp}}$$

$$ROM\;Standard\;error=\sqrt{ROM\;Variance}=\sqrt{\frac{{\left(\frac{SD_{exp}}{Mean_{exp}}\right)}^{2}}{N_{exp}}+\frac{{\left(\frac{SD_{control}}{Mean_{control}}\right)}^{2}}{N_{control}}}$$

We specifically choose this effect size measure to resolve differences between the effect size measures used in the original meta-analyses, in terms of their magnitude and direction of effect, thus allowing these to be pooled. Interestingly, the ROM does not rely on SD, which can differ a lot in preclinical studies and potentially can make other standardised measures like standardised mean differences less reliable. The ROM will represent a relative gain compared with a control group and therefore is only usable to pool data from controlled experiments. To standardise our comparison of the different outcome measures further, we looked at the direction of all the outcome measures and if necessary, transformed this value, to make sure the potential beneficial effect of cell therapy would show a value <1.0. For the cardiac datasets (EF), SCI dataset (neurobehavioural scores), stroke dataset (infarct size reduction) and CKD dataset (blood pressure), an increase in outcome measure indicates a favourable response to cell therapy, so these outcomes where not transformed. The urinary protein measurements (less urinary protein means a favourable response) were converted, using 1/ROM. Thus, throughout this paper, ROM <1.0 indicates a favourable outcome in the therapy group, compared with the control group.

By using ROMs, we provide a common effect size measure across the outcomes in different models. In order to study effect modifiers and explore sources of heterogeneity, variables of interest were:

1. Species, stratified as mice versus rats versus dogs versus sheep versus pigs versus other species.
2. Cell origin, stratified as autologous/syngeneic versus allogeneic versus xenogeneic.
3. Cell type, stratified as bone marrow derived versus mesenchymal stem cells versus blood derived versus tissue- resident cell versus pluripotent cell (e.g. induced pluripotent stem cells (iPSC) or embryonic stem cells (ESC)) versus other cell types.
4. The use of immunosuppression, stratified as drug induced versus genetic.
5. Cell dosage, per kg, corrected through allometric scaling.
6. Delivery method, stratified as peripheral infusion versus local infusion (eg, in a coronary artery, renal artery, cerebral artery) versus direct injection (eg, injection in/around the organ of interest).
7. Tand timing method, stratified as pretreatment versus acute (<24 hours after damage), versus subacute (1–7 days after the damage versus chronic (>7 days after the injury).

For species, we combined gerbils, marmosets and rabbits in one ‘Other’ group, as these species were used in <5 studies in total over all datasets (n in total for this group). We are aware of the important differences between these species and will not draw any conclusions from these groups. For cell type, we combined less frequently used cell types in one ‘other cell type’ variable, including amniotic fluid cells, dermal cells and hair follicle cells. Combinations of different cell types were mentioned separately. In the CSC dataset, multiple tissue-specific cells were used. Only for the univariable analysis we separated these, thereafter they will all be seen as equal tissue-specific cells. To make a comparable variable for dosage across studies, we corrected the variable for the weight of the animal and used allometric scaling. For the different species, we used standardised weights; mice (25 g), rats (300 g). ‘Other’ group (gerbils/marmosets/rabbits) (4000 g), dogs (20 000 g), sheep (12 000 g) and pigs (60 000 g). The dose was divided by the number of grams of the species to the power 0.75 for allometric scaling (number of cells / (weightˆ0.75)). Variables were retrieved from the original publications or recoded if not already present in the desired format.

In our analyses, we included all available data but grouped subsets of data known to be small under one group, unless it was a combination of different cellular products or immunosuppression. We are aware of potential spurious effects in meta-regression when comparing small groups.^12^ However, because the original experimental description coding remains, when performing the multivariable meta-analysis some of these smaller subsets of data re-emerge. For completeness, we judged that it was better to retain them in the datasets but not draw conclusions from them, than to discard them, hence their appearance in some of our graphs and tables.

The search for Phase III trials was performed on 1 November 2019 on the website https://www.clinicaltrials.gov/. Search terms were “stem cells” in combination with the disease of interest (“Renal Failure”, “Kidney Failure”, “Amyotrophic Lateral Sclerosis”, “Motor Neuron Disease”, “Parkinson”, “Alzheimer”, “Huntington”, “Stroke”, “Spinal Cord Injury”, “Myocardial Infarction” and “Heart Failure”). All retrieved studies were screened for their relevance. Trial registration numbers of relevant studies were recorded, regardless of their status.

### Statistical analysis

The variable dose/kg (already corrected for allometric scaling) was log transformed to correct for a non-normal distribution of the data. Random-effects meta-analysis was performed for all datasets. Univariable meta-regression was performed for the chosen variables with the original outcome measure from the study and the new natural log of our generated ROMs and its SE. Natural Logs (of ROMs) were transformed back for proper visualisation and interpretation in figures and tables. In figures, we show the metaregression of univariable analyses of the variable under study. For the dose-response figure, we used metaregression in combination with cubic splines (set to three splines) to properly visualise the association.

Because of known concerns about bias towards no effect with sample sizes for ROMs,^11^ we tested the effect of the variable ‘number of animals’ per comparison on both the original outcomes and ROMs for all datasets. We also summarised the number of animals for the different species in the datasets, to analyse if the variable species could potentially influence ROMs merely through the number of animals used, as one might expect studies in larger animals (and their comparisons within the study) to have smaller sample sizes compared with studies in small animals.

Multivariable analysis with all variables of interest was performed for every dataset individually and for all datasets combined for both original outcomes and ROMs. In the final analysis for all datasets, we corrected for any influence of the datasets themselves by using them as an independent variable. Post hoc testing was performed using a Wald test. Three studies from one dataset (on MI)^8^ were removed for this combined analysis, as these were also present in another dataset.^9^ For the multivariable analyses, p values for the individual variables within the multivariable meta-regression are reported. Although we reported all p values, we will only interpret post hoc p values if the p value for the initial analysis of the variable is significant. A p<0.01 was considered statistically significant to reduce the risk of false-positive testing. Since we are comparing many subgroups, our current analyses can only be interpreted as hypothesis-generating. Statistical analyses were performed using R V.3.1.2^13^ with the additional metafor, lattice, rms and readxl packages.^14–17^ and using Stata V.13.1.^18^

## Results

The combined data from 5 datasets yielded 506 publications with a total of 13 638 included animals. Descriptive statistics of all studies are depicted in table 1.

**Table 1:** Summary of baseline characteristics of included systematic reviews

	Papazova *et al*^5^	Lees *et al*^6^	Antonic *et al*^7^	Jansen Of Lorkeers *et al*^8^	Zwetsloot^9^
Disease type	Nephrology	Stroke	SCI	MI (large animals, all cell types)	MI (all animals, cardiac stem cells)
No of studies	71	117	156	82	80
No of comparisons	31/106*	192	319	125	109
No of animals	1813	2704	5736	1415	1970
Primary outcome	Blood pressure and urinary protein	Infarct volume	Neurobehavioural outcomes	Cardiac function (EF)	Cardiac function (EF)
Overall effect size	0.60 (0.34–0.87)†	24.8% (21.5–28.1)‡	27.3% (25.1% to 29.4%)‡	8.3% (7.1%–9.5%)	10.7% (9.4%–12.1%)

*31 comparisons for blood pressure, 106 for urinary protein.

†Primary outcome for blood pressure converted to a standardised mean difference.

‡Primary outcome for infarct volume and neurobehavioural scores converted to a normalised mean difference.

EF, ejection fraction; MI, myocardial infarction; SCI, spinal cord injury.

The animals used in these studies range from mice and rats, most commonly used in models of CKD, stroke, SCI and MI, to rabbits, gerbils and marmosets in the neurological studies and pigs and sheep predominantly used in models of MI. Regardless of the primary outcome measure used, stem and progenitor cell therapy appeared to improve the outcome in all disease models (table 1). Most common control groups where vehicle treated or PBS treated. Only a minority of papers did not include a proper placebo-treated control group.

To assess the robustness of our chosen effect size measure the ROM, we generated all analyses for the primary outcome (either a raw mean difference, standardised mean difference or normalised mean difference) and compared them to ROMs (see online supplemental tables 1–6). The ROMs generated similar directions of effect and effects of most subgrouping variables compared with the those found with the effect size measures used in the original meta-analyses, deeming ROMs an appropriate effect size measure for our analyses.

### Effect of ROMs and number of animals per comparison

The conversion to ROMs resulted in similar directions of effects of the different variables in our subgroup analyses, compared with the original outcomes from the papers (online supplemental tables 1–5). The number of animals per group was not associated with significant outcomes for ROMs, compared with the originally used outcomes (online supplemental table 7). The number of animals was similar across the variable species (online supplemental table 8).

### Species

#### Univariable analysis

**Figure 1:**
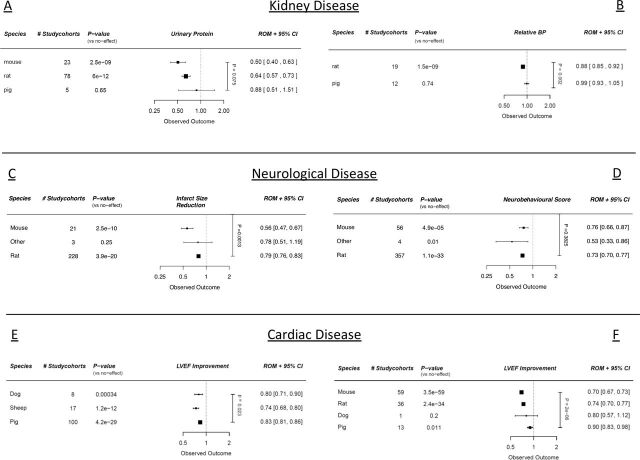
Meta-regression analyses with regard to cell therapy efficacy in different species quantified by (A) urinary protein (CKD), (B) blood pressure (BP) difference (CKD), (C) infarct size (stroke), (D) neurobehavioural scores (SCI), (E) ejection fraction (large animal MI studies), (F) ejection fraction (CSC MI studies). outcomes are expressed in ROMs. Vertical p value represents the total meta-regression. Horizontal p values are significance compared with an assumed ‘no effect’. CKD, chronic kidney disease; CSC, cardiac stem cell; MI, myocardial infarction; ROMs, ratios of means; SCI, spinal cord injury; LVEF, left ventricular ejection fraction.

We stratified our dataset according to species. Small animals such as mice and rats appear to consistently have larger effect sizes across all disease models when analysed with ROMs, compared with larger animals such as dogs, or pigs (figure 1, online supplemental table 1–6). Only the SCI dataset did not show a similar trend (figure 1D) univariably.

#### Multivariable analysis

In the multivariable analyses, species was of no significant importance anymore in most datasets (online supplemental tables 1–6) when analysed with ROMs.

### Cell type

#### Univariable analysis

**Figure 2:**
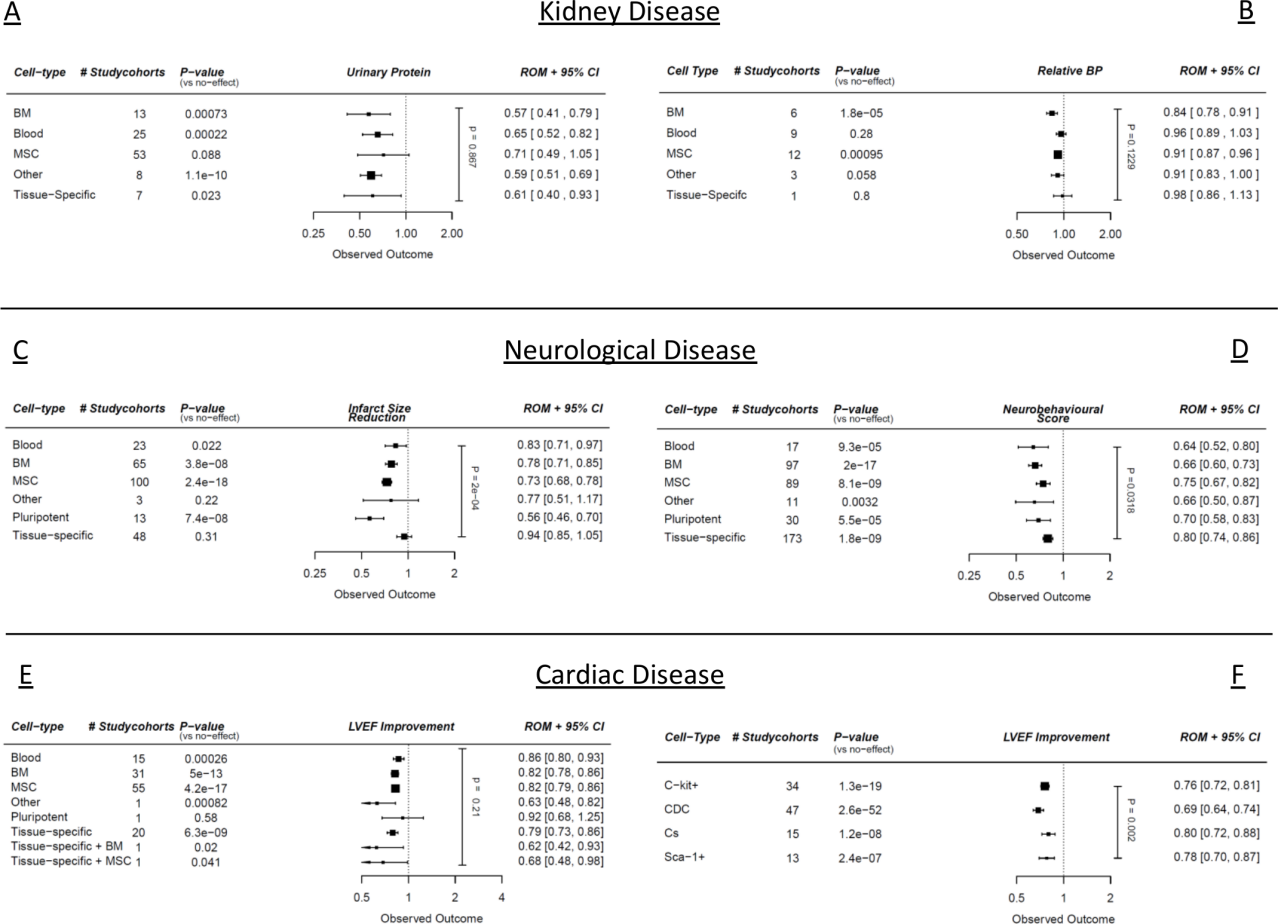
Meta-regression analyses with regard to cell therapy with different cell types quantified by (A) urinary protein (CKD), (B) blood pressure (BP) difference (CKD), (C) infarct size (stroke), (D) neurobehavioural scores (SCI), (E) ejection fraction (large animal MI studies), (F) ejection fraction (CSC MI studies). Outcomes are expressed in ROMs. Vertical p value represents the total meta-regression. Horizontal p values are significance compared with an assumed ‘no effect’. CKD, chronic kidney disease; CSC, cardiac stem cell; MI, myocardial infarction; ROMs, ratios of means; SCI, spinal cord injury; LVEF, left ventricular ejection fraction.

Cell type did not seem to influence any outcome in CKD univariably (p=0.87 and p=0.04, figure 2A, B, online supplemental table 1a and 2a). In the stroke dataset, cell type did explain part of the heterogeneity, with brain-specific cell types performing worse, especially compared with pluripotent cells and mesenchymal stem cells (p=0.0007, figure 2C, online supplemental table 3a). In the SCI dataset, there were no significant differences (p=0.08, figure 2D, online supplemental table 4a). In MI studies in large animals, there was no difference in efficacy between different cell types (p=0.22, figure 2E, online supplemental table 5a). In the MI studies in small animals using CSCs, cardiosphere-derived cells were more effective compared with Sca-1^+^ cells (figure 2F, online supplemental table 6a).

#### Multivariable analysis

In multivariable analyses, there were no significant differences between cell types in all datasets (online supplemental tables 1–6).

### Cell source

#### Univariable analysis

**Figure 3:**
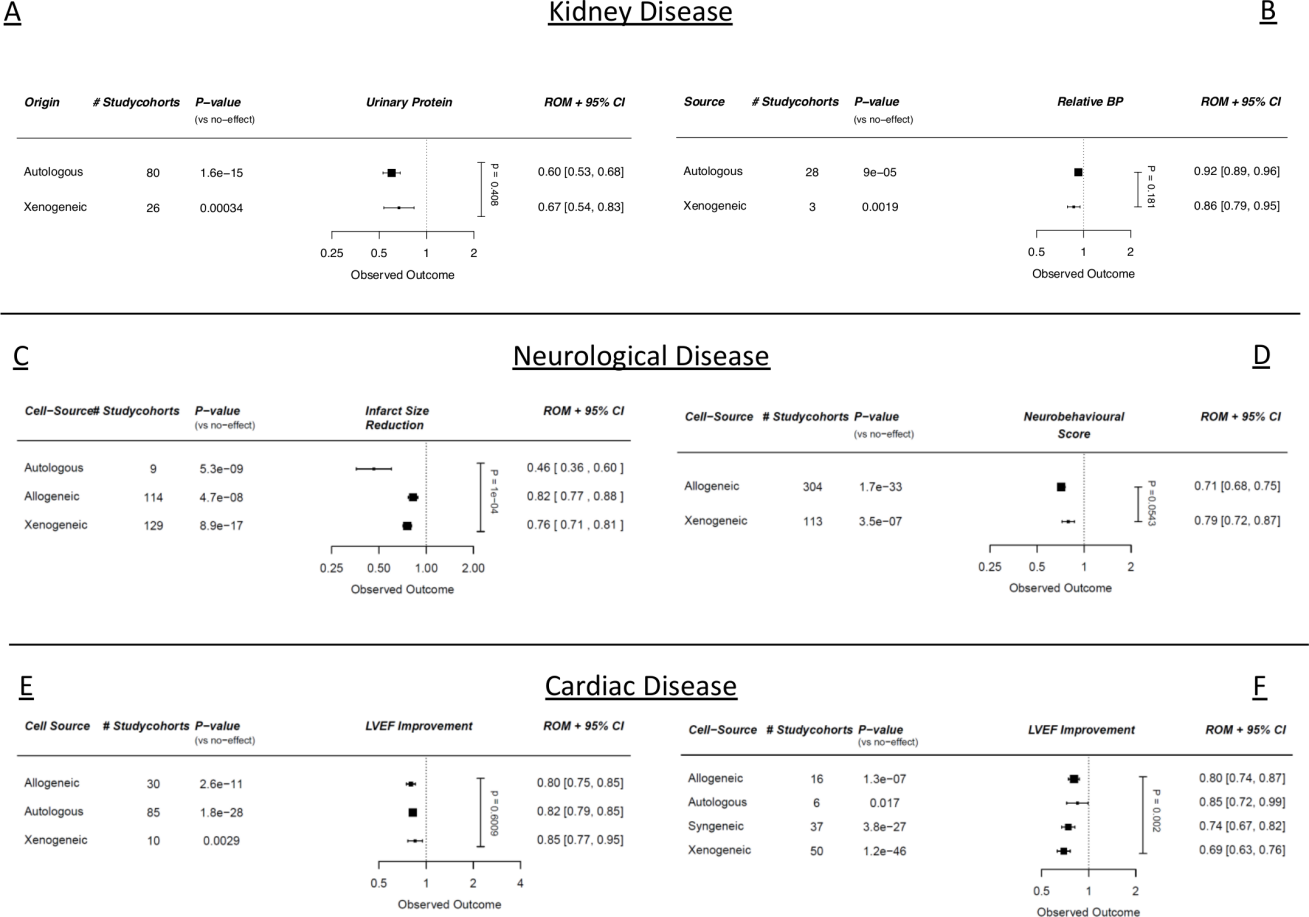
Meta-regression analyses with regard to cell therapy from different cell sources quantified by (A) urinary protein (CKD), (B) blood pressure (BP) difference (CKD), (C) infarct size (stroke), (D) neurobehavioural scores (SCI), (E) ejection fraction (large animal MI studies), (F) ejection fraction (CSC MI studies). Outcomes are expressed in ROMs. Vertical p value represents the total meta-regression. Horizontal p values are significance compared with an assumed ‘no effect’. CKD, chronic kidney disease; CSC, cardiac stem cell; MI, myocardial infarction; ROMs, ratios of means; SCI, spinal cord injury; LVEF, left ventricular ejection fraction.

Cell source did not appear to affect the outcomes in the CKD, SCI and large animal MI datasets (figure 3A, B, D, E). In the stroke data, autologous cells seemed more efficacious compared with allogeneic and xenogeneic stem cells (p=0.0003, figure 3C, online supplemental table 3a). As reported previously,^9^ in the cardiac dataset with CSCs, autologous cells seemed less efficacious compared with the other cell types (p=0.005, figure 3F, online supplemental table 6a).

#### Multivariable analysis

In multivariable analyses, the autologous cells still performed better than the allogeneic and xenogeneic studies in the stroke data only (p=0.0001, online supplemental table 3a).

### Immunosuppression

#### Univariable analysis

**Figure 4:**
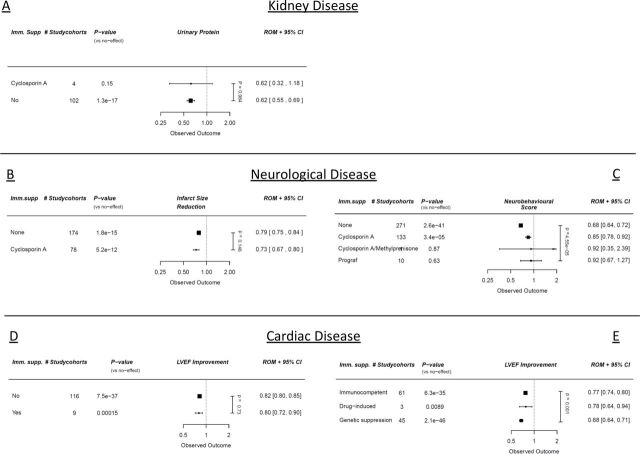
Meta-regression analyses with regard to cell therapy stratified for the use of immunosuppression, quantified by (A) urinary protein (CKD), (B) infarct size (stroke), (C) neurobehavioural scores (SCI), (D) ejection fraction (large animal MI studies), (E) ejection fraction (CSC MI studies). Outcomes are expressed in ROMs. Vertical p value represents the total meta-regression. Horizontal p values are significance compared with an assumed ‘no effect’. CKD, chronic kidney disease; CSC, cardiac stem cell; MI, myocardial infarction; ROMs, ratios of means; SCI, spinal cord injury; LVEF, left ventricular ejection fraction.

The use of ciclosporin A seems to reduce the beneficial effects of stem cell treatment on neurobehavioural scores after SCI (p=0.0005, figure 4C, online supplemental table 4a). In small animal models from the CSC dataset, it seems that stem cells are more effective in genetically modified immunodeficient mice (SCID or athymic mice), compared with immunocompetent animals (p=0.001, figure 4E, online supplemental table 6a). In the other datasets, immunosuppression did not affect treatment efficacy. For the CKD dataset and blood pressure outcomes, there were no studies using any type of immunosuppression.

#### Multivariable analysis

In multivariable analyses, ciclosporin A still showed the same negative effect in the SCI dataset (p=0.002, online supplemental table 4a). In the stroke dataset, ciclosporin A showed the opposite effect in multivariable analysis: the beneficial effect of stem cell treatment was increased, compared with studies when there was no use of immunosuppression (p=0.0005, online supplemental table 3a).

### Cell dose

#### Univariable analysis

**Figure 5:**
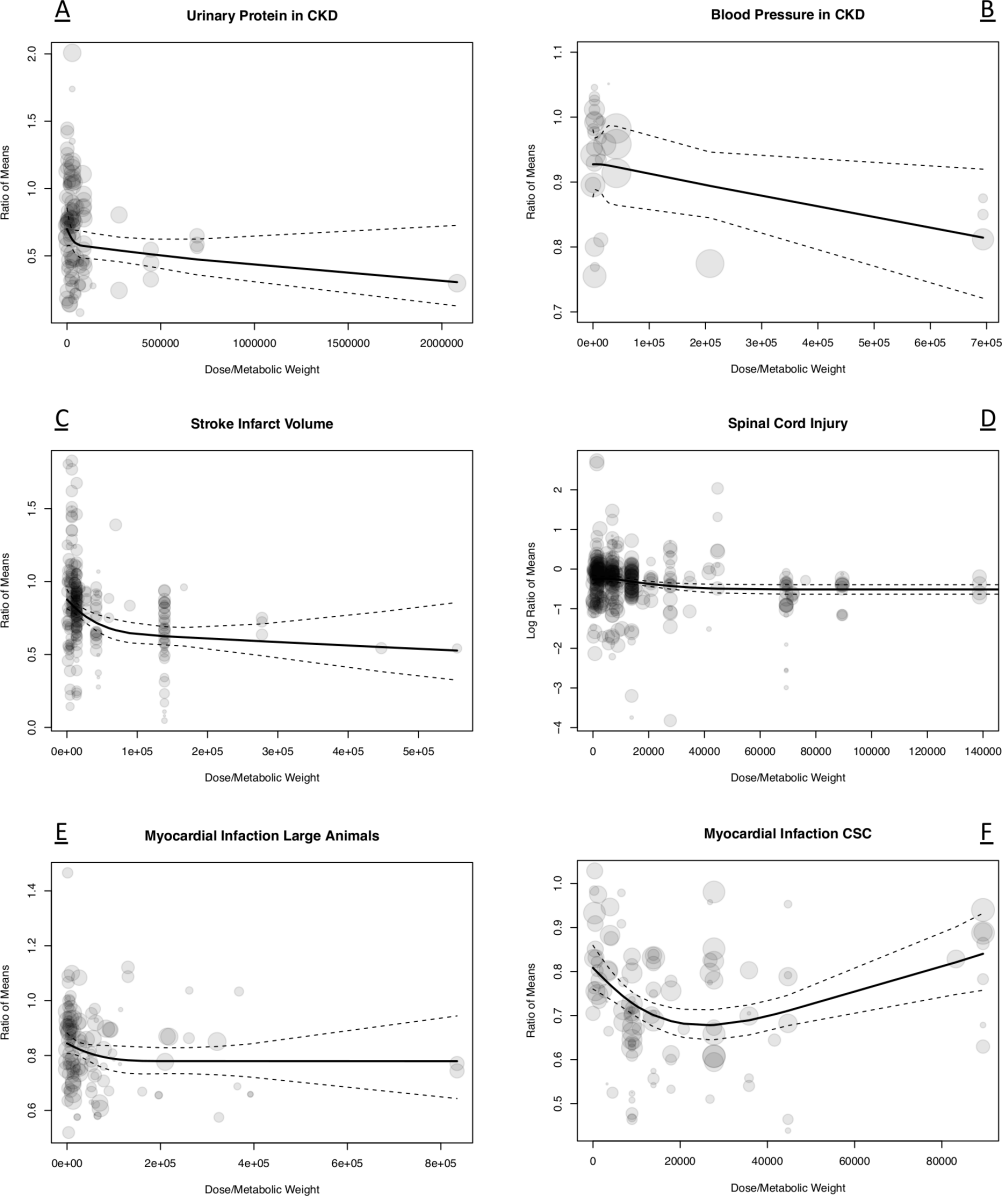
Dose-response curves with regard to cell therapy quantified by (A) urinary protein (CKD), (B) infarct size (stroke), (C) neurobehavioural scores (SCI), (D) ejection fraction (large animal MI studies), (E) ejection fraction (CSC MI studies). outcomes are expressed in the natural log of ROMs. The circles resemble single studies and their size resembles the weighing of the study. The bigger the circle, the smaller the SE of the study. CKD, chronic kidney disease; CSC, cardiac stem cell; MI, myocardial infarction; SCI, spinal cord injury.

In all but the SCI dataset and the CKD blood pressure dataset, higher cell dose (corrected for metabolic weight) was significantly associated with better outcomes (figure 5). In every dataset, the ROM was <1, suggesting similar effects in all datasets with more cells meaning more benefit (figure 5 and online supplemental table 1–6). For the SCI dataset, the graph was more informative without back-transforming the ROM. For clarity, we added the figure with the transformed ROMs as an online supplemental figure 1.

#### Multivariable analysis

In multivariable analyses, cell dose significantly affected the primary outcome in the CKD urinary protein dataset and the stroke dataset. The direction of the effect was still the same (ROM<1), showing better outcomes if more cells were used (table 2).

**Table 2:** P values for all univariable and multivariable analyses of individual datasets and the combination of all datasets

	Papazova *et al*^5^				Lees *et al*^6^		Antonic *et al*^7^		Jansen of lorkeers *et al*^8^		Zwetsloot *et al*^9^		All datasets*	
	Urinary protein		Blood pressure											
	Univar	Multivar	Univar	Multivar	Univar	Multivar	Univar	Multivar	Univar	Multivar	Univar	Multivar	Univar	Multivar
Species	0.08	0.38	**0.005**	0.69	**0.003**	0.06	0.46	0.30	0.03	0.41	**<0.0001**	0.03	**0.0001**	0.70
Cell type	0.87	**0.01**	0.04	0.57	**0.0007**	0.15	0.08	0.33	0.22	0.64	NA	NA	0.09	**0.001**
Cell origin	0.41	0.69	NA	NA	**0.0003**	**0.0001**	0.08	0.25	0.60	0.32	**0.005**	0.61	0.84	0.32
Immunosupp	NA	NA	NA	NA	0.17	**0.0005**	**0.0005**	**0.002**	0.71	0.26	**0.001**	0.41	**0.01**	0.17
Cell dose (/kg)	**0.01**	**0.001**	0.08	0.53	**0.0001**	**0.0002**	0.29	0.11	**0.01**	0.19	**0.01**	0.35	**0.0003**	**0.001**
Time of admin	0.07	0.22	**0.004**	0.73	**<0.0001**	**<0.0001**	0.78	0.30	0.76	0.89	**0.001**	0.92	**0.0003**	**0.0001**
Cell delivery	**0.006**	**0.001**	**0.009**	0.28	**0.003**	0.53	**0.002**	**0.0006**	**0.03**	0.10	0.10	0.68	**0.008**	0.32
(Datasets)													**<0.0001**	**0.0006**

NA, not applicable either because of <5 datapoints in the compared groups, no immunosuppression being used (Papazova *et al*) or because of the use of tissue-resident cells only (Zwetsloot *et al*). Bold significance marks all values ≤0.01.

*With extra correction for the different datasets.

### Delivery strategy

#### Univariable analysis

**Figure 6:**
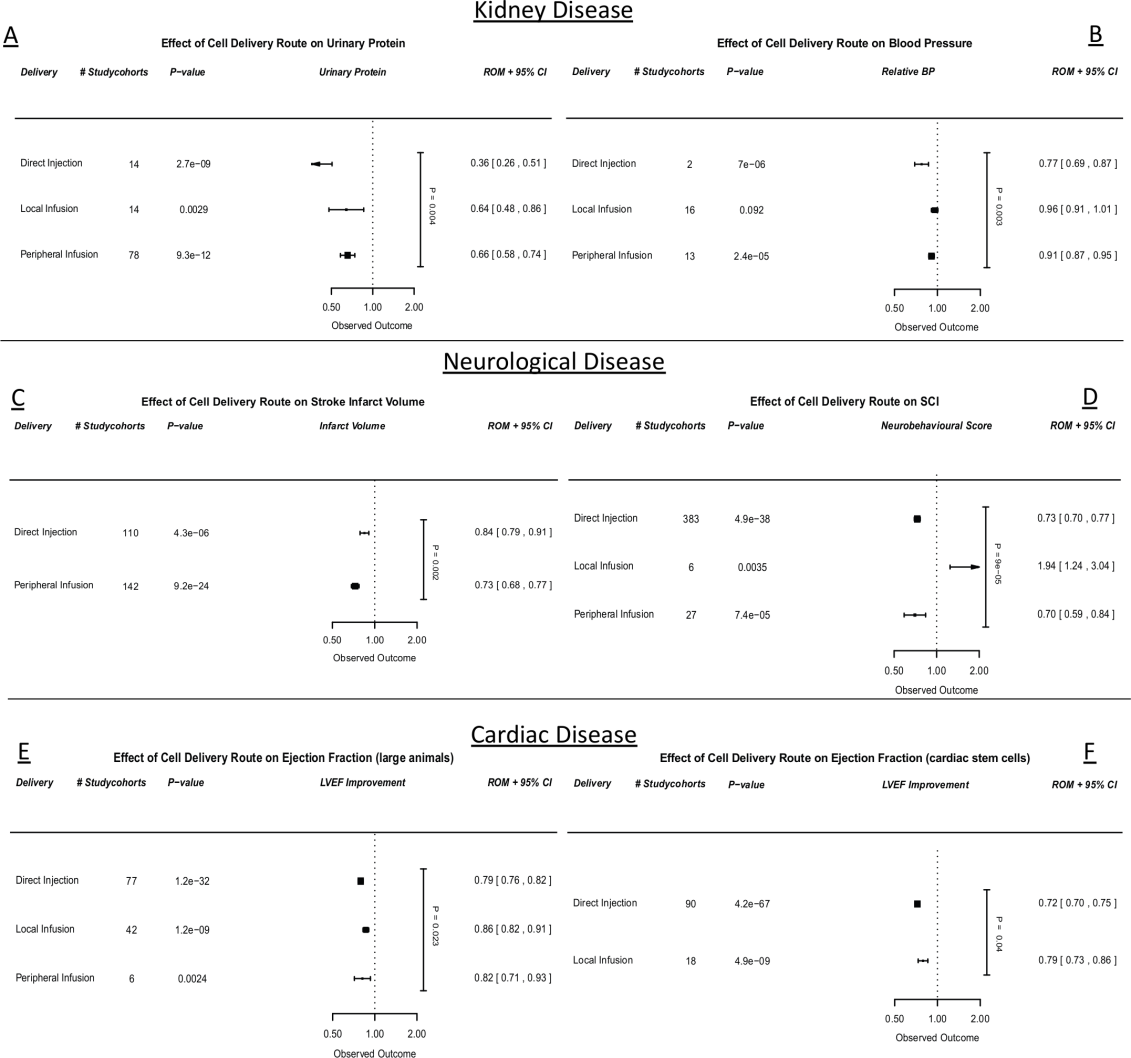
Meta-regression analyses with regard to cell therapy stratified for the use of time of administration, quantified by (A) urinary protein (CKD), (B) infarct size (stroke), (C) neurobehavioural scores (SCI), (D) ejection fraction (large animal MI studies), (E) ejection fraction (CSC MI studies). Outcomes are expressed in ROMs. Vertical p value represents the total meta-regression. Horizontal p values are significance compared with an assumed ‘no effect’. CKD, chronic kidney disease; CSC, cardiac stem cell; MI, myocardial infarction; ROMs, ratios of means.

The mode of delivery influenced stem cell treatment efficacy in most datasets in univariate analysis. In the CKD urinary protein dataset, direct injection was superior over local infusion or peripheral infusion (p=0.01 and p=0.001, respectively, figure 6A, online supplemental table 1a). In the CKD blood pressure dataset, numbers were too small to report an accurate difference between direct injection and other groups, although the comparison reached statistical significance (online supplemental table 2a). In the stroke dataset, peripheral infusion was superior over direct injection (p=0.003, figure 6C, online supplemental table 3a). In the SCI dataset, direct injection and peripheral infusion showed better outcomes compared with local infusion (p<0.001 for both, figure 6D, online supplemental table 4a). In the cardiac large animal dataset, direct injection was superior over local infusion (p=0.01, figure 6E, online supplemental table 5a).

#### Multivariable analysis

In multivariable analyses, cell delivery still significantly influenced the CKD urinary protein dataset and SCI dataset. In the CKD urinary protein, the effect of direct injection was still superior over local or peripheral infusion (p=0.01 and p=0.003 respectively, online supplemental table 1a). In the SCI dataset, direct injection and peripheral infusion remained superior compared with local infusion (p<0.001 and p=0.001, respectively, online supplemental table 4a).

### Time of administration

#### Univariable analysis

**Figure 7:**
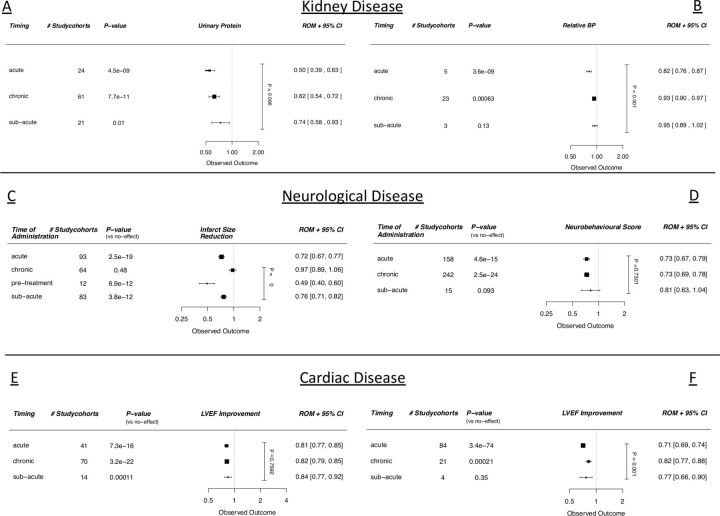
Meta-regression analyses with regard to cell therapy stratified for the use delivery method, quantified by (A) urinary protein (CKD), (B) infarct size (stroke), (C) neurobehavioural scores (SCI), (D) ejection fraction (large animal MI studies), (E) ejection fraction (CSC MI studies). Outcomes are expressed in ROMs. Vertical p value represents the total meta-regression. Horizontal p values are significance compared with an assumed ‘no effect’. BP, blood pressure; CKD, chronic kidney disease; CSC, cardiac stem cell; MI, myocardial infarction; ROMs, ratios of means; SCI, spinal cord injury; LVEF, left ventricular ejection fraction.

Timing of administration of cell therapy significantly affected the outcome in the CKD blood pressure dataset, showing an increased efficacy after acute administration, compared with administration in the chronic setting (p=0.002, figure 7B, online supplemental table 2a). In the stroke dataset, pretreatment was superior over all other administration timings (p<0.001, figure 7C, online supplemental table 3a). Acute and subacute administrations were also better than administration in the chronic setting (p<0.001 and p<0.001, respectively, figure 7C, online supplemental table 3a). In the CSC dataset, acute administration was also associated with better outcomes when compared with administration in the chronic setting (figure 7F, online supplemental table 6a).

#### Multivariable analysis

In multivariable analysis, time of administration still impacted the outcome significantly (p<0.0001) in the stroke dataset, showing a greater effect of pretreatment with cell therapy compared with either acute, subacute or chronic administration (online supplemental table 5a).

### Analysis of the combined datasets

#### Univariable analysis

In univariable analysis, mice and rats are performing significantly better compared with pigs (p=0.001 for both). Also, mice performed better than rats (p=0.001). There was so significant difference for cell type (p=0.09), cell origin (p=0.09), cell origin (p=0.84) or immunosuppression (p=0.01). Higher dose was significantly associated with better outcomes (p=0.0003). For delivery strategies, direct injection and peripheral infusion performed better than local infusion (p=0.002 and p=0.001, respectively). For time of administration, pretreatment performed better than chronic administration and subacute administration (p=0.001 and p<0.001, respectively). There were significant differences between the different datasets univariably (see online supplemental table 9).

#### Multivariable analysis

The multivariable analysis of all datasets combined revealed that cell dose (p=0.001), cell type (p=0.001) and time of administration (p=0.0001) share significant effects on stem cell treatment efficacy in all datasets. For cell dose, this means the higher the dose, the better the effect of cell therapy regardless of the disease model (p=0.001). For cell type, this was apparent for tissue-specific cells, being less efficacious compared with pluripotent cells (p=0.006), bone marrow-derived cells (p=0.001) and mesenchymal stem cells (p=0.001). For time of administration, pretreatment with cell therapy resulted in greater benefits compared with administration in the acute, subacute or chronic setting (=0.0001). Furthermore, also the different datasets showed a significant effect on our outcome measure (p=0.0006), with the CKD Blood pressure dataset performing worse compared with the CKD urinary protein dataset (p=0.001), the CSC dataset (p=0.01) and the SCI dataset (=0.01). The SCI dataset performed better compared with the stroke dataset (p=0.003). The estimated ROMs and confidence intervals are provided in online supplemental table 9.

### Current phase III trials in stem and progenitor cell therapy

Since we wanted to know the stage in which the different cell therapy fields are right now, we searched for all phase III trials in each disease. Our search retrieved 34 phase III trials, of which 25 were in the cardiac field (table 3). The other nine trials are in different neurological diseases. No phase III studies for kidney diseases were found through our search.

**Table 3:** Summary of all current cell therapy clinical trials in phase III in the fields of nephrology, neurology and cardiology (www.clinicaltrials.gov search on 1 November 2019)

Disease type	# Phase III studies	Trial codes
**Nephrology**		
Renal failure	0	
Kidney failure	0	
**Neurodegenerative disorders**		
Amyotrophic lateral sclerosis/motor neuron disease	2	**NCT01933321**, NCT03280056
Parkinson’s disease	1	NCT03128450
Alzheimer’s disease	0	
Huntington’s disease	0	
**Acute neurological disorders**		
Stroke	3	NCT01716481, NCT02849613, NCT03545607
Spinal cord injury	3	NCT01676441, **NCT01873547**, NCT03935724
**Cardiac disease**		
Myocardial infarction	16	**NCT00279175**, **NCT00316381**, NCT00350766, **NCT00363324**, NCT00501917, NCT00684060, NCT00725738, NCT00950274, **NCT01187654**, **NCT01392105**, NCT01394432, NCT01569178, NCT01652209, NCT02323620, **NCT02672267**, NCT03404063,
Heart failure	9	**NCT00333827**, **NCT00462774**, **NCT00743639**, **NCT00747708**, **NCT00841958**, NCT01693042, NCT01759212, NCT02032004, **NCT02248532**

Bold=completed. Normal font=ongoing/unknown status

Out of all of these studies, 14 have been registered as completed (2 in neurological diseases, 12 in cardiac diseases), while two studies were terminated early. The other 28 studies are either at the stage of recruiting patients, or have already passed their completion date, but have not updated their status on ClinicalTrials.gov.

## Discussion

In this paper, we show using meta-analysis that experimental design choices lead to outcome trends that are universal across preclinical studies of stem and progenitor cell therapy in models of kidney, neurological and cardiac disease. Attention to these choices is needed to optimise research in this field and ensure realistic expectations of tested therapeutics. This analysis should be considered hypothesis generating. Nevertheless, p values are provided to help researchers prioritise future hypothesis testing.

Species most strikingly affected the efficacy of cell therapy in univariable analyses, with decreasing treatment efficacy going from rodents to larger mammals. There was no important effect of species on our outcomes when multivariable analysis was applied, arguing that choice of species might be correlated with multiple other factors that influence cell therapy efficacy. Large animal trials have an important part to play in the evaluation of new therapies destined for human use. It is generally argued that larger animals more closely resemble human anatomy, physiology and ultimately reaction to therapy.^4^ Comparing our univariable and multivariable analyses, we cannot conclude whether it is the actual animal size or other factors that come with large animal experiments that affects efficacy. Large animal studies might be associated with different experimental choices with regard to physiological monitoring and ethically mandated interventions, therapeutic delivery and cell dose. Furthermore, it has been proposed that large animal studies resemble human clinical trials better with regard to blinding, randomisation and susceptibility to bias, although to our knowledge, this has never been proven. A theoretical limitation of using ROM is a potential bias towards detecting no effect when study sizes are small.^11^ However, as we could find no evidence for a relationship between ROM and number of animals per experimental cohort for this (online supplemental table 7), or our other comparisons, we do not believe that our finding reflects limited sampling in studies using larger species.

Interestingly, in almost all datasets, tissue-specific cell types (eg, a progenitor cell that resides in the organ of interest) did not show clear benefits over first generation cells like bone marrow-derived cells, mesenchymal stem cells, circulating cells or pluripotent cells, which is in line with current perspectives.^19 20^ In our multivariable analysis of all datasets combined, tissue-specific cells even showed less efficacy compared with other cell products, although this result might be predominantly driven by the neurological datasets. Of note, the modes of action might also be different for the different cell types under study, as a paracrine cell and a self-integrating ‘residential’ cell type could benefit a damaged organ differently. In the cardiac field, multiple preclinical studies hinted on superior effects of combinations of progenitor cell types, hypothetically making use of multiple cell-specific abilities.^21–24^

In all these different disease models, stem and progenitor cell therapy seems to provide a comparable gain of function, regardless of cell origin. As shown previously in the cardiac field, xenogeneic cells might show less benefit than allogeneic or autologous/syngeneic cell sources.^25^ Allogeneic and autologous cells have shown comparable benefits,^25^ with the same proposed mechanisms, as is confirmed by our analyses.

Immunosuppression has shown to affect treatment efficacy in the datasets on stroke and SCI. In cardiac disease, cyclosporine has also been proposed as an agent that might affect both disease outcome and cell therapy.^26^ However, in our combined datasets and multivariable analyses, we could not confirm a common effect of drug-induced immunosuppression, which was also not seen when directly studied on tissue-specific CSCs.^27^ Interestingly, recent data argues that the primary effect of cardiac cell therapy might be due to a provoked immunological reaction, regardless of the cell type and regardless of any functionality of the cell therapy.^28^ This would partially explain the comparable outcomes between all different cell types. The fact that immune suppression negatively influenced cell therapy in the neurological studies might also be explained by this phenomenon.

Cell dose showed the same beneficial effect across multiple datasets and showed a significant effect in the combined multivariable analysis. Although an increase in treatment effect with increasing dose was to be expected, it remains challenging in both large animal experiments and clinical settings to grow billions of cells for a single treatment. To our knowledge, this is the first time that a direct effect of cell dose is shown across multiple diseases. The route of cell delivery, whether it was local or peripheral, did not affect treatment efficacy in any of the disease models. For timing strategies, only pretreatment with cell therapy was associated with a better response across all datasets, which poses a challenge from a translational perspective for the majority of diseases studied.

The factor ‘dataset’, added to the multidataset analysis to correct for confounding between dataset and other variables, showed a significant difference between some datasets. While correcting for this factor in our multivariable analysis, this suggests that there might be some disease-specific heterogeneity for cell therapy or collinearity between other variables that cannot be grasped in general analyses as the ones conducted in this paper. Luckily, most variables of interest showed comparable directions of effect in both the single datasets and the general analysis, suggesting similar and generalisable effects across diseases.

### Limitations

Since this is an analysis using existing data, we are dependent on the quality of the initial primary datasets and their included studies. The included datasets have shown high risks of bias within primary studies and publication bias.^5–9^ This means that these bias are likely also present in our current analysis, which might lead to overestimation of effects and is to be taken into consideration when interpretating these results.

We should be careful with the interpretation of our results, as effect modification through known and unknown variables might cause significance without true causal relevance. For example, some effects are reduced when correcting for other variables of interest, such as the effect of immunosuppression and cell origin in the MI-CSC dataset. Moreover, differences in the magnitude of effect sizes between the different disease model datasets and the possibility that this reflects differences in the availability of therapeutic targets amenable to stem cell therapy, need to be investigated to determine if this explains why the variable ‘dataset’ accounted for significant heterogeneity in our analyses.

In our study, testing for interactions between variables was not part of our analysis plan. Nevertheless, with hindsight, we can see circumstances where this might have been of value. For example, differential interactions between the type of stem cell used and degree of inflammatory change induced by different models might explain the lack of consistency of influence of immunomodulatory adjunct therapies. Similarly, we have not attempted to correct for prevalence of randomisation, blinding or other surrogates of experimental quality.

Of note, the nature of experimental controls used across the datasets varied considerably, ranging from no (placebo) procedure at all, to vehicle-solution delivery or other ‘non-therapeutic cell’ administration. Such variability is also expected to contribute to heterogeneity and subsequent noise in the combined datasets.

For this study, the use of ROMs was essential to provide a common denominator for comparisons across sometimes very different field-specific outcome measures. As noted earlier, despite a theoretical bias towards no effects when study sizes are small, we were unable to detect such a correlation in our analysis. Moreover, our transformed univariable ROM outcomes agreed well with the general direction of effects reported in the original publications investigation of heterogeneity.

### Stem and progenitor cell therapy research in different stages of research

Table 3 summarises ongoing and completed phase III trials for stem and progenitor cell therapy in fields of nephrology, neuroscience (both neurodegenerative and acute disease), MI and heart failure. Although we found clear effects of stem and progenitor cell therapy and common factors affecting all these different diseases, some research areas are already reaching clinical endpoints, while others are still in the process of passing preclinical phases. Reasons for this discrepancy remain to be elucidated, although factors like interest and funding might play a role. Current success stories in other disease entities, include cellular retinal transplants^29^ and application of stem and progenitor cells or newly generated cartilage in degenerated joints,^30^ offer hope for the diseases studied here.

### What can we learn from this?

In light of the questioned translatability of rodent models in cell therapy research,^31^ these current analyses confirmed the decrease in effect size across multiple disease models, when increasing animal size, as reported previously for the heart. Cell source and cell type only marginally influence efficacy, while immunosuppressive effects cannot be generalised to all diseases studied. Why the effects are more pronounced for brain diseases is unclear. This is the first time that we so clearly show an important dose response in all these diseases combined, suggesting that large numbers of cells are needed in human disease for any effect. The fact that pretreatment is most beneficial is of little clinical value, as no disease under study will wait to manifest itself for a pretreatment to be initiated.

These data emphasises the need for a clear understanding of the translational potential of different animal models and highlights the importance of calls for standardisation of animal models and therapeutic approaches in all research areas, for which only the neurological and cardiac fields have started first initiatives.^32–35^ This study illustrates the importance of cross-domain systematic review and meta-analysis as tools for understanding important commonalities of value in future preclinical and clinical experimental design.

## Data Availability

Data are available in a public, open access repository. All online supplemental tables and figure can be viewed and accessed on the open repository Figshare, https://doi.org/10.6084/m9.figshare.13778626.v1 and https://doi.org/10.6084/m9.figshare.13779001.v1. All data that were used from the existing datasets is available on the open repository Figshare, https://doi.org/10.6084/m9.figshare.13670050.v1. The code for our analysis (performed in R and Stata) is available on Figshare, https://doi.org/10.6084/m9.figshare.13670113.v1
